# Low-dose aspirin, statins, and metformin and survival in patients with breast cancers: a Norwegian population-based cohort study

**DOI:** 10.1186/s13058-023-01697-2

**Published:** 2023-08-30

**Authors:** L. Lukas Löfling, Nathalie C. Støer, Bettina Kulle Andreassen, Giske Ursin, Edoardo Botteri

**Affiliations:** 1 Department of Research, Cancer Registry of Norway, Postboks 5313 Majorstuen, 0304 Oslo, Norway; 2 Cancer Registry of Norway, Oslo, Norway; 3 Department of Nutrition, Institute of Basic Medical Sciences, University of Oslo, Oslo, Norway; 4 Department of Preventive Medicine, University of Southern California, Los Angeles, USA; 5 Section for Colorectal Cancer Screening, Cancer Registry of Norway, Oslo, Norway

**Keywords:** Breast cancer, Low-dose aspirin, Statins, Metformin, Survival, Cohort, Population-based

## Abstract

**Background:**

Previous studies assessed the prognostic effect of aspirin, statins, and metformin in breast cancer (BC) patients, with inconclusive results.

**Methods:**

We performed a nationwide population-based cohort study to evaluate if post-diagnostic use of low-dose aspirin, statins, and metformin was associated with BC-specific survival. Women aged ≥ 50 years and diagnosed with BC in 2004–2017, who survived ≥ 12 months after diagnosis (follow-up started 12 months after diagnosis), were identified in the Cancer Registry of Norway. The Norwegian Prescription Database provided information on prescriptions. Multivariable Cox proportional hazard models were used to estimate hazard ratios (HR) and 95% confidence intervals (CI) for the association between post-diagnostic use and BC-specific survival, overall and by oestrogen receptor (ER) status.

**Results:**

A total of 26,190 patients were included. Of these, 5324 (20%), 7591 (29%), and 1495 (6%) were post-diagnostic users of low-dose aspirin, statins, and metformin, respectively. The median follow-up was 6.1 years, and 2169 (8%) patients died from BC. HRs for use, compared to no use, were estimated at 0.96 (95% CI 0.85–1.08) for low-dose aspirin (ER+: HR = 0.97, 95% CI 0.83–1.13; ER−: HR = 0.97, 95% CI 0.73–1.29, *p* value for interaction = 0.562), 0.84 (95% CI 0.75–0.94) for statins (ER+: HR = 0.95, 95% CI 0.82–1.09; ER−: HR = 0.77, 95% CI 0.60–1.00, *p* value for interaction = 0.259), and 0.70 (95% CI 0.51–0.96) for metformin (compared to use of non-metformin antidiabetics) (ER+: HR = 0.67, 95% CI 0.45–1.01; ER−: HR = 1.62, 95% CI 0.72–3.62, *p* value for interaction = 0.077).

**Conclusion:**

We found evidence supporting an association between post-diagnostic use of statins and metformin and survival, in patients with BC. Our findings indicate potential differences according to ER status.

**Supplementary Information:**

The online version contains supplementary material available at 10.1186/s13058-023-01697-2.

## Background

Globally, breast cancer (BC) is the leading cancer-related cause of death among women [1], accounting for approximately 700,000 deaths in 2020. Survival for patients with BC has improved in the last decades due to more accurate diagnostic procedures and tailored treatment strategies, and the introduction of new and effective systemic therapies [2, 3]. However, the prognosis of some types of BCs remains poor, such as advanced stage BC or triple negative BC (TNBC), because of their unfavourable biology and lack of targeted therapies [4]. Hence, new treatment strategies are urgently needed.

Aspirin (acetylsalicylic acid) in low-doses and statins are frequently used for treatment and prevention of cardiovascular conditions [5, 6], and metformin is the first line pharmacological treatment for diabetes mellitus type II with mild to moderate hyperglycaemia [7]. Several plausible biological mechanisms suggest that aspirin, statins, and metformin could reduce the risk of BC and improve its prognosis [8–10]. While randomised clinical trials assessing the possible prognostic effect of statins in patients with BC is lacking, the trials assessing the effect of aspirin and metformin have reported that adding these medications to standard treatment did not improve prognosis [11–13]. Epidemiological studies are still valuable in the presence of published clinical trials. Compared to clinical trials, epidemiological studies evaluate the effect of medications in clinical practice, and they tend to be less selective in the constitution of the study population and have longer follow-up time. Several epidemiological studies have assessed the possible prognostic effect of aspirin [14–20], statins [21–29], and metformin [30–35] in patients with BC, and mainly reported no association or association with improved prognosis. These previous clinical trials and epidemiological studies have often been impaired by inclusion of small study populations, use of self-reported data on medication use, and a lack of analyses stratified by tumour and patient characteristics, such as stage, molecular subtype, and age at diagnosis. This has made it difficult to assess the possible prognostic effects of those medications in depth. Therefore, we performed a large nationwide population-based cohort study of patients diagnosed with BC, where we aimed to explore a possible prognostic effect of use of low-dose (≤ 160 mg) aspirin, statins, and metformin in patients with BC, analysed as a whole and stratified by several tumour and patient characteristics such as age at diagnosis, molecular features, stage, and use of chemotherapy.

## Methods

### Data sources

In this study, we linked individual level data from the Cancer Registry of Norway [36], the Norwegian Prescription Database [37], the Cause of Death Registry [38], the National Population Registry [39], and sociodemographic data from Statistics Norway [40]. The linkage was performed using the 11-digit unique personal identification number assigned to all Norwegian residents at birth or immigration [41]. This linkage has previously been used to assess the association between use of non-cancer medications and survival in patients with cancer, including BC [42–44].

#### Cancer Registry of Norway

In 1952, the Cancer Registry of Norway started recording detailed information on each cancer diagnosed in Norway [36]. The completeness of the Cancer Registry of Norway is estimated 99%. The topography and morphology codes from the International Classification of Diseases for Oncology, 3^rd^ revision (ICD-O-3) were used to classify the cancers. The ICD-O-3 morphology code was used to categorise histology as ductal carcinoma (code 850), lobular carcinoma (code 852), other forms of carcinoma (code: 801–823, 825–849, 851, 853–867, 894), and non-carcinoma. Information on oestrogen receptor (ER) status (ER+, ER−, missing), progesterone receptor (PR) status (PR−, PR+, missing), and human epidermal growth factor receptor 2 (HER2) status (overexpressed HER2 [HER2 +], not overexpressed HER2 [HER2-], missing) is routinely retrieved from pathology reports and registered by the Cancer Registry of Norway [4]. From February 2012 onwards, the threshold for ER + changed from 10 to 1% reactivity. PR + tumours were defined as tumours with ≥ 10% reactivity throughout the study period. Since 2012, Ki-67 (reported as a percentage of Ki-67 positive tumour cells) has been registered routinely by the Cancer Registry of Norway. The molecular subtype was defined using the registry information on receptor status (ER, PR, HER2) and Ki-67: luminal A (ER + and/or PR + , HER2-, Ki-67 ≤ 14), luminal B HER2- (ER + and/or PR + , HER2-, Ki-67 > 14), luminal B HER2 + (ER + and/or PR + , HER2 +), HER2 + (ER−, PR−, HER2 +), and TNBC (ER-, PR-, HER2-) [44]. If missing Ki-67, tumour grade I (low) was used to define a tumour as luminal A and II–III (intermediate–high) to define it as luminal B HER2-. The definition from the National Cancer Institute’s Surveillance, Epidemiology, and End Results (SEER) Program was used to categorise the disease stage as localised, regional, or distant [45]. Patients categorised as users of chemotherapy by the Cancer Registry of Norway include both patients treated with chemotherapy and patients planned to be treated with chemotherapy. From now on they will be referred to as chemotherapy users.

#### Norwegian prescription database

Data on prescribed medications were provided by the Norwegian Prescription Database. Since 2004, the database collects, mandated by law, detailed individual level information on all prescribed medications dispensed from community pharmacies in Norway [37]. The database includes information on the date of dispensation, Anatomical Therapeutic Chemical (ATC) code for the dispensed medication, strength (i.e., amount of active pharmaceutical ingredient per unit, e.g., mg per tablet), and the number of defined daily doses (DDD; i.e., the average maintenance dose per day for a medication used for its main indication in adults [46]) per dispensation.

#### Other registries

Information on cause and date of death were provided by the Cause of Death Registry [38]. The cause of death is recorded using the International Classification of Diseases, 10^th^ revision (ICD-10) codes. The National Population Registry provided information on migration [39], and Statistics Norway provided information on marital status, education, number of children, and country of origin, which is based on country of birth going back up to two generations from the individual [40, 47].

### Study population

For this population-based cohort study, all women residing in Norway born between 1925 and 1986 diagnosed with primary invasive BC (ICD-O-3 topography code: C50) between July 2004 and December 2017 were identified in the Cancer Registry of Norway. First, inclusion was limited to patients with carcinoma (i.e., we excluded, for example, sarcomas, lymphomas, and carcinoids). Second, the inclusion was limited to patients living at least 6 months in Norway prior to the BC diagnosis, to ensure that all patients were covered by the registries for a minimum period prior to the diagnosis. Third, the inclusion was limited to patients with no history of invasive cancer (except non-melanoma skin cancer; ICD-O-3 topography code: C44). Fourth, the inclusion was limited to patients aged ≥ 50 years at diagnosis. As women aged < 50 years rarely use low-dose aspirin, statins and metformin, the limitation to patients aged ≥ 50 years was applied to obtain more comparable age distributions in users and non-users, and to obtain a homogeneous population of mainly postmenopausal women. Finally, follow-up started 12 months after their BC diagnosis, thus patients who died or emigrated before that were excluded.

### Assessment of medication use

The use of medications was assessed using the information in the Norwegian Prescription Database on dispensation of prescribed medications [37]. The specific medications were identified using the ATC codes (Additional file 1: Table S1). Use of medications was assessed, separately for each medication, in the time from 1 month after diagnosis until end of follow-up. The assessment started 1 month after diagnosis to avoid changes in use of medications in the time surrounding diagnosis and surgery. Post-diagnostic use was defined as dispensation of ≥ 270 DDD of the specific medication. To avoid immortal time bias, medication use was handled as time-varying as follows: Patients contributed person-time to the no use group of a specific medication in the time from start of follow-up (i.e., 12 months after diagnosis) until the date they fulfilled the definition of post-diagnostic use of that specific medication (≥ 270 DDD) or until end of follow-up. Patients contributed person-time to the use group from the date they fulfilled the definition of post-diagnostic use of that specific medication until end of follow-up. If a patient fulfilled the definition of post-diagnostic use prior to the start of follow-up (i.e., 12 months after diagnosis), then they contributed person-time to the use group of that specific medication from the start of follow-up (Additional file 1: Fig. S1).

### Statistical analysis and study design

The follow-up for each patient in the cohort started at 12 months after their BC diagnosis and ended at the date of death due to BC (the event of interest, ICD-10: C50), death due to other causes, emigration, or administrative censoring (31st December 2018), whichever occurred first. Cox proportional hazard models, with time since start of follow-up (12 months after diagnosis) as the underlying time scale, were used to estimate hazard ratios (HR) and 95% confidence intervals (CI) for the association between post-diagnostic use of low-dose (≤ 160 mg) aspirin, statins, and metformin and BC-specific survival, and overall survival. Variables included to adjust the estimates from the Cox proportional hazard models were; post-diagnostic use of medications (i.e., non-aspirin antiplatelets, non-metformin antidiabetics, non-steroidal anti-inflammatory drugs, beta-blockers, angiotensin converting enzyme inhibitors, angiotensin receptor blockers, calcium channel blockers, and diuretics [For the ATC codes, see Additional file 1: Table S1]), age at diagnosis (continuous), highest attained education in the year prior to diagnosis (none/primary school, secondary school, university), marital status in the year prior to diagnosis (not married/not in partnership, married/in partnership), number of children in the year prior to diagnosis (0, 1, 2, ≥ 3), country of origin (Norway, other Nordic countries [i.e., Sweden, Denmark, Finland, and Iceland], rest of the world), cancer stage at diagnosis (localised, regional, distant, missing), molecular subtype (luminal A, luminal B HER2-, luminal B HER2 + , HER2 + , TNBC, missing), histology (ductal carcinoma, lobular carcinoma, other carcinomas). The estimates for low-dose aspirin, statins, metformin were mutually adjusted for each other. Missing information for any covariate was handled by including a separate missing category in the variable. The association was analysed in the overall BC population and stratified by ER status (+ , −), age at diagnosis (50–69.9 years, ≥ 70 years), molecular subtype (luminal A, luminal B HER2-, luminal B HER2 + , HER2 + , TNBC), stage (localised, regional, distant), and use of chemotherapy (yes, no). The interaction between the exposure (post-diagnostic use of low-dose aspirin, statins, and metformin) and the patient and tumour characteristics were assessed by introducing an interaction term between the exposure variable and the variable for the specific characteristic.

The reference groups for low-dose aspirin and statins were no use of the specific medication. While the reference group for metformin was use of non-metformin antidiabetics, this was done to address confounding by indication. If a medication is indicated for treatment of a condition associated with the outcome, then the most suitable reference group is use of other medications used to treat the same condition [48]. Metformin users have diabetes mellitus type II, which is associated with increased risk of BC-related death [49], hence, to address confounding by indication, the reference group was users of other antidiabetics. For low-dose aspirin and statins we do not have any sensible active comparator groups, and the conditions treated with low-dose aspirin and statins are not clearly associated with the risk of BC-related death.

Schoenfeld residuals were used to investigate the proportional hazards assumption. All tests were two-sided with a 5% significance level. All data management and statistical analyses were performed using R version 4.2.1 (http://cran.r-project.org).

#### Sensitivity analyses

To assess the influence of the definition of post-diagnostic use and when follow-up starts, in a separate analysis, follow-up started at 6 months after diagnosis (including patient diagnosed between July 2004 and June 2018, and excluding patients who died or emigrated in the first 6 months after diagnosis), and the definition of post-diagnostic use was set to ≥ 100 DDDs. To remove the influence of pre-diagnostic use of the medications, in a separate analysis, incident users only were included (i.e., all patients with at least one dispensation of the specific medication within 6 months prior to the diagnosis were excluded). In addition, to assess the influence of the reference group in the analysis of metformin use, the reference group was changed from use of non-metformin antidiabetics to any no use of metformin. Finally, in a separate analysis, peri-diagnostic use (at least one dispensation of the specific medication within 3 months prior to the diagnosis) was applied as the exposure definition. In this analysis, patients diagnosed between July 2004 and December 2018 were included, and follow-up started at the date of diagnosis.

## Results

A total of 37,735 women were diagnosed with a first time primary invasive BC in Norway between July 2004 and December 2017 (Additional file 1: Fig. S2). Of these, we excluded women with non-carcinoma BC (n = 237), less than 6 months residency in Norway prior to their BC diagnosis (n = 96), invasive cancer diagnosis (excluding non-melanoma skin cancer) prior to their BC diagnosis (n = 2350), age < 50 years (n = 7997), and those who died or emigrated within the first 12 months after their BC diagnosis (n = 865). In total, 26,190 women with BC were included, and during a median follow-up of 6.1 years 5324 (20%), 7591 (29%), and 1495 (6%) were post-diagnostic users of low-dose aspirin, statins, and metformin, respectively, and 2169 (8%) patients died from BC. Users of low-dose aspirin and statins, compared to non-users, were older at diagnosis, less educated, and more often used other medications (Table 1). Users of metformin, compared to users of other antidiabetics, were younger and more often diagnosed with localised disease (Table 1). Of the included patients, 47% have missing information on chemotherapy use.

**Table 1:** Baseline characteristics of patients with breast cancer by post-diagnostic use of low-dose aspirin, statins, and metformin, Norway 2004–2017

	No low–dose aspirin (N = 20,866)	Low–dose aspirin (N = 5324)	No statins (N = 18,599)	Statins (N = 7591)	Non–metformin antidiabetics (N = 313)	Metformin (N = 1495)
*Age (years) at diagnosis*						
Median (Q1, Q3)	62.0 (55.0, 68.0)	68.0 (62.0, 75.0)	62.0 (55.0, 69.0)	65.0 (60.0, 72.0)	69.0 (62.0, 77.0)	65.0 (59.0, 72.0)
*Education*						
None/primary school	4977 (23.9%)	1715 (32.2%)	4423 (23.8%)	2269 (29.9%)	108 (34.5%)	540 (36.1%)
Secondary school	10,142 (48.6%)	2644 (49.7%)	8923 (48.0%)	3863 (50.9%)	161 (51.4%)	740 (49.5%)
Higher	5747 (27.5%)	965 (18.1%)	5253 (28.2%)	1459 (19.2%)	44 (14.1%)	215 (14.4%)
*Marital status*						
Not married/partnered	8616 (41.3%)	2420 (45.5%)	7931 (42.6%)	3105 (40.9%)	155 (49.5%)	691 (46.2%)
Married/partnered	12,250 (58.7%)	2904 (54.5%)	10,668 (57.4%)	4486 (59.1%)	158 (50.5%)	804 (53.8%)
*Number of children*						
0	2451 (11.7%)	546 (10.3%)	2204 (11.9%)	793 (10.4%)	46 (14.7%)	179 (12.0%)
1	2931 (14.0%)	785 (14.7%)	2689 (14.5%)	1027 (13.5%)	56 (17.9%)	225 (15.1%)
2	8565 (41.0%)	2038 (38.3%)	7594 (40.8%)	3009 (39.6%)	89 (28.4%)	528 (35.3%)
≥ 3	6919 (33.2%)	1955 (36.7%)	6112 (32.9%)	2762 (36.4%)	122 (39.0%)	563 (37.7%)
*Country of origin*						
Norway	18,979 (91.0%)	4969 (93.3%)	16,893 (90.8%)	7055 (92.9%)	279 (89.1%)	1312 (87.8%)
Other Nordic countries^a^	598 (2.9%)	136 (2.6%)	554 (3.0%)	180 (2.4%)	7 (2.2%)	39 (2.6%)
Rest of the world	1289 (6.2%)	219 (4.1%)	1152 (6.2%)	356 (4.7%)	27 (8.6%)	144 (9.6%)
*Stage*						
Local	12,859 (61.6%)	3319 (62.3%)	11,331 (60.9%)	4847 (63.9%)	151 (48.2%)	888 (59.4%)
Regional	6296 (30.2%)	1621 (30.4%)	5642 (30.3%)	2275 (30.0%)	127 (40.6%)	497 (33.2%)
Distant	553 (2.7%)	95 (1.8%)	542 (2.9%)	106 (1.4%)	12 (3.8%)	33 (2.2%)
Missing	1158 (5.5%)	289 (5.4%)	1084 (5.8%)	363 (4.8%)	23 (7.3%)	77 (5.2%)
*Molecular subtype*						
Luminal A	4600 (22.0%)	1053 (19.8%)	4029 (21.7%)	1624 (21.4%)	45 (14.4%)	275 (18.4%)
Luminal B HER2-	9081 (43.5%)	2189 (41.1%)	8057 (43.3%)	3213 (42.3%)	138 (44.1%)	681 (45.6%)
Luminal B HER2 +	1689 (8.1%)	360 (6.8%)	1511 (8.1%)	538 (7.1%)	22 (7.0%)	108 (7.2%)
HER2 +	777 (3.7%)	170 (3.2%)	707 (3.8%)	240 (3.2%)	10 (3.2%)	44 (2.9%)
TNBC	1420 (6.8%)	335 (6.3%)	1273 (6.8%)	482 (6.3%)	21 (6.7%)	97 (6.5%)
Missing	3299 (15.8%)	1217 (22.9%)	3022 (16.2%)	1494 (19.7%)	77 (24.6%)	290 (19.4%)
*Histology*						
Ductal carcinoma	16,482 (79.0%)	4174 (78.4%)	14,674 (78.9%)	5982 (78.8%)	255 (81.5%)	1214 (81.2%)
Lobular carcinoma	2667 (12.8%)	664 (12.5%)	2379 (12.8%)	952 (12.5%)	38 (12.1%)	162 (10.8%)
Other carcinoma	1717 (8.2%)	486 (9.1%)	1546 (8.3%)	657 (8.7%)	20 (6.4%)	119 (8.0%)
*Concomitant medications*						
Low-dose aspirin	–	–	1849 (9.9%)	3475 (45.8%)	145 (46.3%)	634 (42.4%)
Other antiplatelets	185 (0.9%)	411 (7.7%)	93 (0.5%)	503 (6.6%)	25 (8.0%)	61 (4.1%)
Statins	4116 (19.7%)	3475 (65.3%)	–	–	196 (62.6%)	973 (65.1%)
Metformin	861 (4.1%)	634 (11.9%)	522 (2.8%)	973 (12.8%)	–	–
Non-metformin antidiabetics	370 (1.8%)	363 (6.8%)	238 (1.3%)	495 (6.5%)	–	–
NSAIDs	3111 (14.9%)	972 (18.3%)	2650 (14.2%)	1433 (18.9%)	32 (10.2%)	301 (20.1%)
Beta-blockers	2611 (12.5%)	2023 (38.0%)	2142 (11.5%)	2492 (32.8%)	114 (36.4%)	555 (37.1%)
ACE-inhibitors	1360 (6.5%)	972 (18.3%)	1082 (5.8%)	1250 (16.5%)	87 (27.8%)	297 (19.9%)
ARB	4612 (22.1%)	2307 (43.3%)	3789 (20.4%)	3130 (41.2%)	129 (41.2%)	758 (50.7%)
CCB	2782 (13.3%)	1749 (32.9%)	2319 (12.5%)	2212 (29.1%)	127 (40.6%)	556 (37.2%)
Diuretics	4259 (20.4%)	2448 (46.0%)	3591 (19.3%)	3116 (41.0%)	166 (53.0%)	756 (50.6%)

*Q* quartile, *HER2* human epidermal growth factor receptor 2, *TNBC* triple negative breast cancer, *NSAIDs* non-steroidal anti-inflammatory drugs, *ACE* angiotensin converting enzyme, *ARB* angiotensin receptor blocker, *CCB* calcium channel blocker

^a^Sweden, Denmark, Finland, Iceland

### Use of low-dose aspirin, statins and metformin and breast cancer-specific survival

**Fig. 1:**
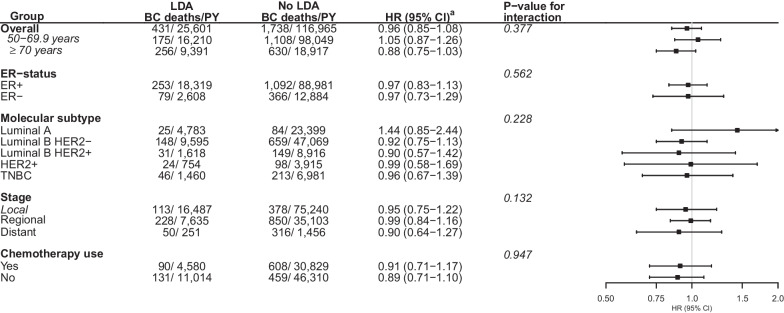
Association between post-diagnostic use (dispensation of ≥ 270 defined daily doses after diagnosis) of low-dose aspirin, compared to no use, and breast cancer-specific survival, Norway 2004–2017, by age, molecular subtype, stage, and use of chemotherapy. *Abbreviation* Low-dose aspirin (LDA), breast cancer (BC), person-years (PY), hazard ratio (HR), confidence interval (CI), oestrogen receptor (ER), human epidermal growth factor receptor 2 (HER2), triple negative breast cancer (TNBC). ^a^Adjusted for age at diagnosis, education, marital status, number of children, country of origin, stage, molecular subtype, histology, and post-diagnostic use of concomitant medications (statins, metformin, non-aspirin antiplatelets, non-metformin antidiabetics, non-steroidal anti-inflammatory drugs, beta-blockers, angiotensin converting enzyme inhibitors, angiotensin receptor blockers, calcium channel blockers, and diuretics). The stratified estimates are adjusted for all variables except for the specific stratification variable

HR for the association between use of low-dose aspirin, compared to no use, and BC-specific survival was estimated at 0.96 (95% CI 0.85–1.08) (ER+: HR = 0.97, 95% CI 0.83–1.13; ER−: HR = 0.97, 95% CI 0.73–1.29, *p* value for interaction = 0.562) (Fig. 1). We found an indication of an association between use of low-dose aspirin and longer BC-specific survival in patients aged ≥ 70 years at diagnosis (HR = 0.88, 95% CI 0.75–1.03) (Table 1, Additional file 1: Fig. S3).

**Fig. 2:**
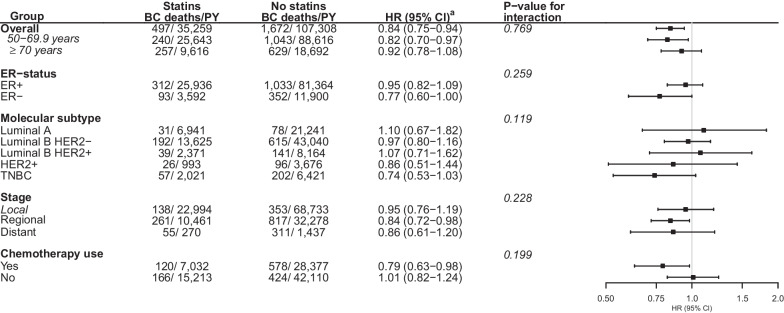
Association between post-diagnostic use (dispensation of ≥ 270 defined daily doses after diagnosis) of statins, compared to no use, and breast cancer-specific survival, Norway 2004–2017, by age, molecular subtype, stage, and use of chemotherapy. *Abbreviation* Breast cancer (BC), person-years (PY), hazard ratio (HR), confidence interval (CI), oestrogen receptor (ER), human epidermal growth factor receptor 2 (HER2), triple negative breast cancer (TNBC). ^a^Adjusted for age at diagnosis, education, marital status, number of children, country of origin, stage, molecular subtype, histology, and post-diagnostic use of concomitant medications (low-dose aspirin, non-aspirin antiplatelets, metformin, non-metformin antidiabetics, non-steroidal anti-inflammatory drugs, beta-blockers, angiotensin converting enzyme inhibitors, angiotensin receptor blockers, calcium channel blockers, and diuretics). The stratified estimates are adjusted for all variables except for the specific stratification variable

For the association between use of statins, compared to no use, and BC-specific survival, the HR was estimated at 0.84 (95% CI 0.75–0.94) (ER+: HR = 0.95, 95% CI 0.82–1.09; ER−: HR = 0.77, 95% CI 0.60–1.00, *p* value for interaction = 0.259) (Fig. 2, Additional file 1: Fig. S4). An association with longer BC-specific survival was found among patients aged < 70 years (HR = 0.82, 95% CI 0.70–0.97), patients with regional disease (HR = 0.84, 95% CI 0.72–0.98), and chemotherapy users (HR = 0.79, 95% CI 0.63–0.98). In addition, an indication of an association with longer BC-specific survival was found among the patients with TNBC (HR = 0.74, 95% CI 0.53–1.03).

**Fig. 3:**
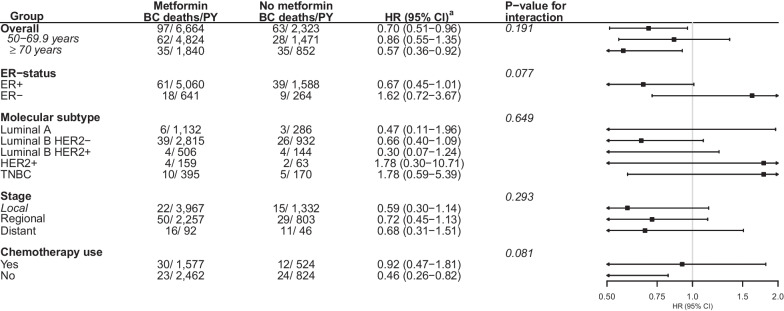
Association between post-diagnostic use (dispensation of ≥ 270 defined daily doses after diagnosis) of metformin, compared to use of non-metformin antidiabetics, and breast cancer-specific survival, Norway 2004–2017, by age, molecular subtype, stage, and use of chemotherapy. *Abbreviation* Breast cancer (BC), person-years (PY), hazard ratio (HR), confidence interval (CI), oestrogen receptor (ER), human epidermal growth factor receptor 2 (HER2), triple negative breast cancer (TNBC). ^a^Adjusted for age at diagnosis, education, marital status, number of children, country of origin, stage, molecular subtype, histology, and post-diagnostic use of concomitant medications (low-dose aspirin, non-aspirin antiplatelets, statins, non-steroidal anti-inflammatory drugs, beta-blockers, angiotensin converting enzyme inhibitors, angiotensin receptor blockers, calcium channel blockers, and diuretics). The stratified estimates are adjusted for all variables except for the specific stratification variable

The HR for the association between metformin use, compared to use of non-metformin antidiabetics, and BC-specific survival was estimated at 0.70 (95% CI 0.51–0.96) (ER+: HR = 0.67, 95% CI 0.45–1.01; ER−: HR = 1.62, 95% CI 0.72–3.62, *p* value for interaction = 0.077) (Fig. 3, Additional file 1: Fig. S5). An association with longer BC-specific survival was found among patients aged ≥ 70 years (HR = 0.57, 95% CI 0.36–0.92) and those not using chemotherapy (HR = 0.46, 95% CI 0.26–0.82).

### Sensitivity and secondary analyses

The estimated HRs for the association between use, compared to no use, and overall survival were 1.12 (95% CI 1.04–1.21) for low-dose aspirin, 0.84 (95% CI 0.78–0.91) for statins, and 0.79 (95% CI 0.64–0.96) for metformin (compared to use of non-metformin antidiabetics).

In the sensitivity analyses where follow-up started at 6 months after diagnosis and ≥ 100 DDDs was used as the definition of post-diagnostic use, 27,894 patients with BC were included. Of these, 6697 (24%), 8870 (32%), and 1839 (7%) were post-diagnostic users of low-dose aspirin, statins, and metformin, respectively. Of the patients included in the incident user analyses, 2187 (10%), 2898 (14%), and 604 (2%), were incident users of low-dose aspirin, statins, and metformin, respectively. The results in these two sensitivity analyses, and in the analysis for metformin where the reference group was changed from use of non-metformin antidiabetics to any no use of metformin, were in line with the results in the main analysis (Additional file 1: Figs. S5–S11). In the peri-diagnostic use analyses (at least one dispensation of the specific medication within 3 months prior to the diagnosis), 29,600 patients with BC were included, 3202 (11%), 4862 (16%), and 939 (3%) were peri-diagnostic users of low-dose aspirin, statins, and metformin, respectively. The estimated HRs for the association between use, compared to no use, and BC-specific survival were 1.11 (95% CI 0.99–1.25) for low-dose aspirin, 0.90 (95% CI 0.81–1.01) for statins, and 1.19 (95% CI 0.85–1.67) for metformin (compared to use of non-metformin antidiabetics) (Additional file 1: Figs. S12–S14).

## Discussion

### Main findings

In this large population-based cohort of patients with BC, we found evidence supporting an association between post-diagnostic use of statins and metformin and survival (both breast cancer-specific and overall), the findings indicate that there might be differences in association by ER status. We found no clear evidence supporting an association between post-diagnostic use of low-dose (≤ 160 mg) aspirin and survival.

### Interpretation and comparison with other studies

Statins decrease levels of serum cholesterol by inhibiting hydroxymethylglutaryl-coenzyme A reductase (HMG-CoAR), an enzyme involved in the biosynthesis of mevalonate acid in the mevalonate pathway of cholesterol synthesis [9]. Statins have been suggested to exert anti-cancer effect through different pathways resulting in inhibition of cellular proliferation, induction of apoptosis, and suppression of tumour cell migration. Randomised clinical trials investigating the prognostic effect of statins in BC patients is lacking. A Danish phase III trial (Clinicaltrials.gov identifier: NCT04601116) started in 2021 and aims to recruit 3360 women with ER + BC, with the objective of evaluating the effect of statins on breast cancer prognosis (with invasive disease-free survival as the primary outcome measure). A few epidemiological studies have investigated the association between post-diagnostic use of statins and BC-specific survival, with inconclusive results [25–29]. Consistent with our findings, some epidemiological studies report an association between post-diagnostic use of statins and a decreased risk of BC-specific death [25, 27, 28], while others report no such association [26, 28]. Based on registry data on 15,140 Scottish patients with BC, Mc Menamin et al. [26] reported no association between post-diagnostic use of statins and risk of BC-specific death (HR = 0.93, 95% CI 0.77–1.12); however, an association with pre-diagnostic use of statins (HR = 0.85, 95% CI 0.74–0.98) was reported. Nowakowska et al. [28] included 23,192 patients with BC identified in the Texas Cancer Registry and reported an association between post-diagnostic use of statins and a decreased risk of BC-specific death for patients with TNBC (HR = 0.42, 95% CI 0.20–0.88), but not for patients with non-TNBC (HR = 0.97, 95% CI 0.71–1.39). This is consistent with our findings in TNBC and non-TNBC patients.

The biological reason behind the association between post-diagnostic use of statins and a decreased risk of BC-specific death in patients with TNBC but not in patients with other molecular subtypes remains unclear. However, patients with TNBC receive chemotherapy more often than patients with other types of BC, and pre-clinical studies have suggested that statins exert a therapeutic effect through enhancing the effect of chemotherapeutic agents [50, 51].

To corroborate the hypothesis of a potential interaction between statins and chemotherapeutic agents, we estimated a decreased risk of BC-specific death associated with use of statins among recipients of chemotherapy (HR = 0.79, 95% CI 0.63–0.98) but not among patients who do not receive chemotherapy (HR = 1.01, 95% CI 0.82–1.24) (Fig. 2).

Metformin functions by reducing resistance to insulin and decreasing serum levels of insulin [10]. Pre-clinical studies have suggested that metformin inhibits cancer progression and prognosis via direct effects on the cancer cells, by acting on the AMP-activated protein kinase (AMPK)/mammalian target of rapamycin (mTOR) pathway, and indirect effects by decreasing serum levels of insulin and insulin-like growth factor 1 (IGF-1). The association between post-diagnostic use of metformin and BC-specific survival has been studied in a small number of epidemiological studies [31, 32, 34]. Our finding of a decreased risk of BC-specific death associated with post-diagnostic use of metformin corroborates both pre-clinical studies and previous epidemiological studies evaluating the association in patients with both BC and diabetes mellitus type II [10, 31, 32, 34]. Kim et al. [34] evaluated 386 South Korean diabetic patients with BC and reported a decreased risk of BC-specific death associated with post-diagnostic use of metformin, compared to non-metformin antidiabetics, in patients with ER + and/or PR + BC but not in patients with BC with both ER- and PR-. The association with a decreased risk of BC-specific death among metformin users with ER +/PR + BC but not with ER − and PR − BC is consistent with our finding of an association in patients with ER + BC only. Furthermore, it corroborates the hypothesis that the AMPK/mTOR pathway plays a role in the development of resistance to endocrine therapy in ER + BC and that the metformin activity on the AMPK/mTOR pathway can re-sensitise the ER + BCs to endocrine therapy [52]. In conflict with our findings, a randomised clinical trial published in 2022 by Goodwin et al. [13], including 3649 BC patients without diabetes, reported that addition of metformin to standard treatment did not improve invasive disease-free survival. The estimate did not differ by ER status. The Goodwin trial confirmed the findings from previously published smaller randomised clinical trials [12].

There are plausible mechanisms suggesting that aspirin affect BC progression and prognosis by altering levels of prostaglandins [8]. Aspirin inhibits cyclooxygenase (COX), an enzyme involved in the biosynthesis of prostaglandins, which are mediators of inflammation and pain. Prostaglandins are suggested to promote cellular proliferation and invasiveness, and stimulate the activity of aromatase, an enzyme responsible for the biosynthesis of oestrogens, which are drivers of ER +/luminal BC. In addition, prostaglandins are the precursors of thromboxane, which is required to facilitate platelet aggregation, and the antiplatelet effect of aspirin has been suggested to inhibit tumour cells from initiating metastases. The results reported by epidemiological studies assessing the prognostic effect of the post-diagnostic use of aspirin (including both low-dose and regular dose) are inconclusive [14–20]. Our findings of no association corroborate a number of previous epidemiological studies [17–20], as well as the recently finished randomised phase III Aspirin after Breast Cancer (ABC) trial [11], which included 3021 BC patients and reported that addition of aspirin (300 mg) to the standard treatment did not improve disease-free survival. In contrast, some epidemiological studies have reported an association with longer BC-specific survival [14–16]. Using data from the Iowa Women’s Health Study (591 patients with BC), Blair et al. [14] reported that post-diagnostic use of aspirin was associated with a decreased risk of BC-specific death (HR = 0.53, 95% CI 0.30–0.93). Similarly, Holmes et al. [15] reported that use of aspirin after diagnosis was associated with a decreased risk of BC-specific death (2–5 days a week: HR = 0.29, 95% CI 0.16–0.52; 6–7 days a week: HR = 0.36, 95% CI 0.24–0.54) in 4164 patients with BC participating in the Nurses’ Health Study. The results did not differ when stratified by stage, menopausal status, body mass index, or ER-receptor status. In addition, a Scottish registry study by Fraser et al. (4627 patients with BC) [16] reported an association between post-diagnostic use of aspirin and a decreased risk of BC-specific death (HR = 0.53, 95% CI 0.45–0.63). The strength or dose of aspirin may have been inconsistent between studies. Similar to our study, all the epidemiological studies that reported no association included almost exclusively low dose-aspirin users [17–20], while neither the Iowa Women’s Health Study nor the Nurses’ Health Study restricted their questionnaires to users of low-dose aspirin, nor did they collect information on the dose of aspirin for the surveys used in the studies by Blair et al. and Holmes et al. [14, 15]. Aspirin in higher doses is rarely used in Norway [53], but more frequently used in the USA [54]. Therefore, it is possible that the Iowa Women’s Health Study and the Nurses’ Health Study included a non-neglectable proportion of users of aspirin in higher doses. One hypothesis is that high doses of aspirin are necessary to see an effect on BC prognosis.

Breast cancer treatments, such as chemotherapy, have well-documented cardio-toxic side effects, potentially leading to increased risk of all-cause deaths (driven by cardiovascular related deaths). Giving medications frequently used to prevent or treat cardiovascular diseases in addition to the chemotherapy might prevent these side effects, resulting in prolonged overall survival. Previous studies assessed the association for statins, metformin, and aspirin with overall survival in patients with BC, have been conflicting, reporting no association and associations with both longer and shorter overall survival [14, 16, 18, 19, 25–28, 31, 32]. However, most of the previous studies, in line with our study, have reported that post-diagnostic use of statins and metformin were associated with a decreased risk of all-cause death. In contrast with most studies, we found that use of low-dose aspirin was associated with an increased risk of all-cause death, potentially due to an increased risk of cardiovascular-related deaths among the users of low-dose aspirin.

### Strengths and limitations

The main strength of our cohort study is the population-based design with data from nationwide registries of high quality and completeness, minimising the risk of misclassification bias and selection bias. The use of a prescription database avoided self-reported use of drugs, which may be less accurate and are associated with a higher risk of introducing misclassification bias. Another strength was the large sample size and the detailed information on tumour characteristics that allowed for exploring the potential prognostic effect in depth.

However, there are several limitations in our study that needs mentioning. First, the Norwegian Prescription Database records information on filled prescriptions but it contains no information on actual use or adherence, and the database only includes information on dispensed medications from community pharmacies and not medications given at hospitals or nursing homes. This may have resulted in some misclassification of both users and non-users, possibly leading to underestimation of the associations between use of the medications and survival (HR biased towards 1). Second, patients contributed person time to the user group of a specific medication from the date they fulfilled the criteria of post-diagnostic use until end of follow-up. This definition of exposure might not capture the real time-dependent exposure. By handling medication use in this way we presumed that the potential prognostic effect would last after discontinuation for patients who discontinued the medication before end of follow-up. If this presumption does not hold, then the estimated difference in survival between users and no users would be smaller than the true difference between the groups, and the estimated associations between post-diagnostic use and survival would have been underestimated (HR biased towards 1). Third, we did not have access to information on comorbid conditions. However, this was addressed by using the information on dispensed medications as proxy for comorbid conditions. Fourth, considering the high proportion of patients with missing information on chemotherapy use, the results stratified by chemotherapy use should be interpreted with caution. Fifth, that we did not use active comparators for low-dose aspirin and statins may have introduced bias through confounding by indication. However, the fact that the conditions treated with low-dose aspirin and statins are not clearly associated with the risk of BC-related death may somewhat alleviate this concern. Sixth, we missed information on important confounders, such as the body mass index. This may have biased the results. For example, if high body mass index is associated with increased use of the medications studied and an increased risk of BC-related death, not including body mass index possibly resulted in a bias towards an increased HR. Finally, some of the subgroups have few events, making it difficult to draw conclusions form these subgroups.

## Conclusion

In our large nationwide population-based cohort study, we found evidence supporting an association of post-diagnostic use of statins and metformin with survival in patients with BC. The findings indicate that there might be differences in association by ER status. We found no clear evidence supporting an association for post-diagnostic use of low-dose aspirin.

### Supplementary Information

**Additional file 1.** Supplementary material.

## Data Availability

Due to Norwegian law, we are not allowed to make the data publicly available. However, the data can be requested from the registry holders.
