# Supplementary material for: Low-dose aspirin, statins, and metformin and survival in patients with breast cancers: a Norwegian population-based cohort study
Source: Breast Cancer Res. 2023 Aug 30;25:101. doi: 10.1186/s13058-023-01697-2 (PMC10466817; doi:10.1186/s13058-023-01697-2)
Supplement: Supplementary file 1 — Additional file 1. Supplementary material. [file 13058_2023_1697_MOESM1_ESM.docx]

**Supplementary material**

| **Supplementary Table 1** Medications and corresponding Anatomical Therapeutic Chemical (ATC) codes | |
| --- | --- |
| **Medications** | **ATC code** |
| Low-dose aspirin | B01AC06, -56 |
| Statins | C10AA, C10BA02, -05, -06 |
| Metformin (mono- or combination therapy) | A10BA02, A10BD03, -05, -07, -08, -10, -11, -15, -20, -23 |
| Non-aspirin antiplatelets | B01AC01-05, B01AC7-30 |
| Non-metformin antidiabetics | A10A, A10BA01, -03, A10BB, A10BC, A10BD01, -04, -06, -09, -12, -19, -21, -24, A10BF, A10BG, A10BH, A10BJ, A10BK, A10BX |
| Non-steroidal anti-inflammatory drugs | M01A |
| Beta-blockers | C07 |
| Angiotensin-converting enzyme inhibitors | C09A, C09B |
| Angiotensin receptor blockers | C09C, C09D |
| Calcium channel blockers | C08, C09BB, C09DB |
| Diuretics | C03, C07B, C09BA, C09DA |


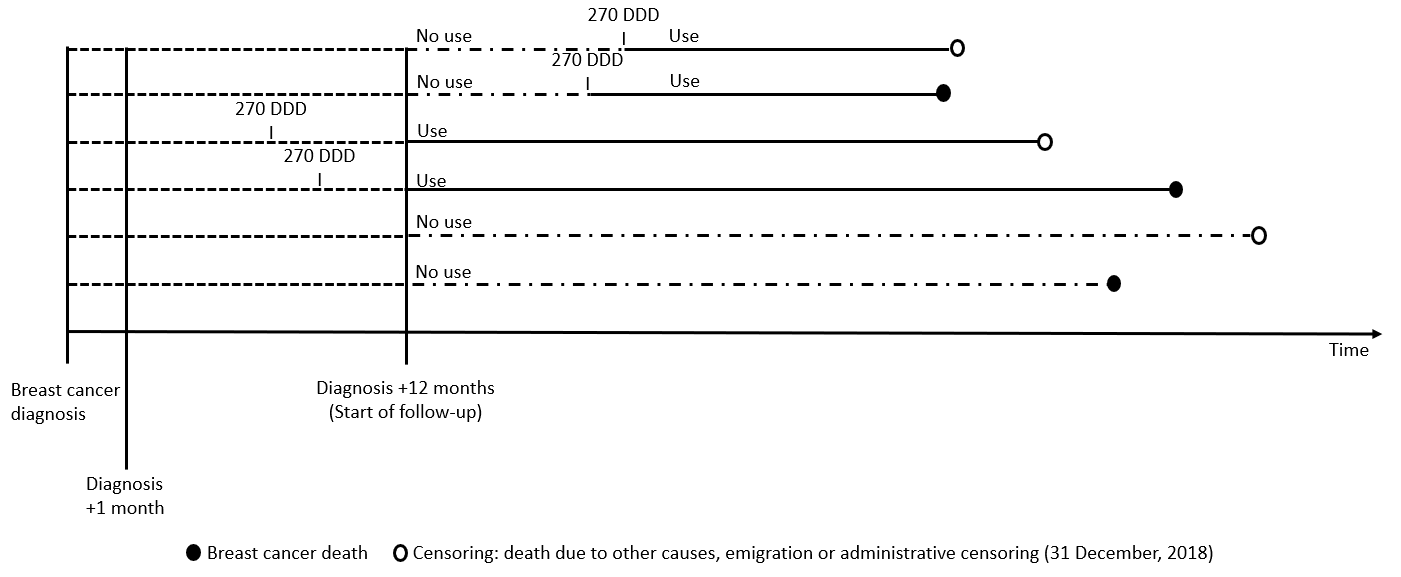


**Supplementary Figure 1** Assessment of medication use and follow-up

*Abbreviations;* DDD: defined daily doses


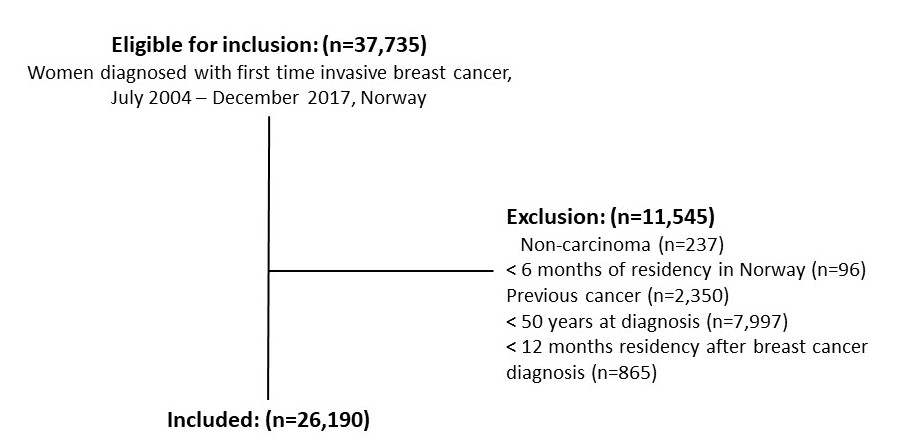


**Supplementary Figure 2** Flow chart


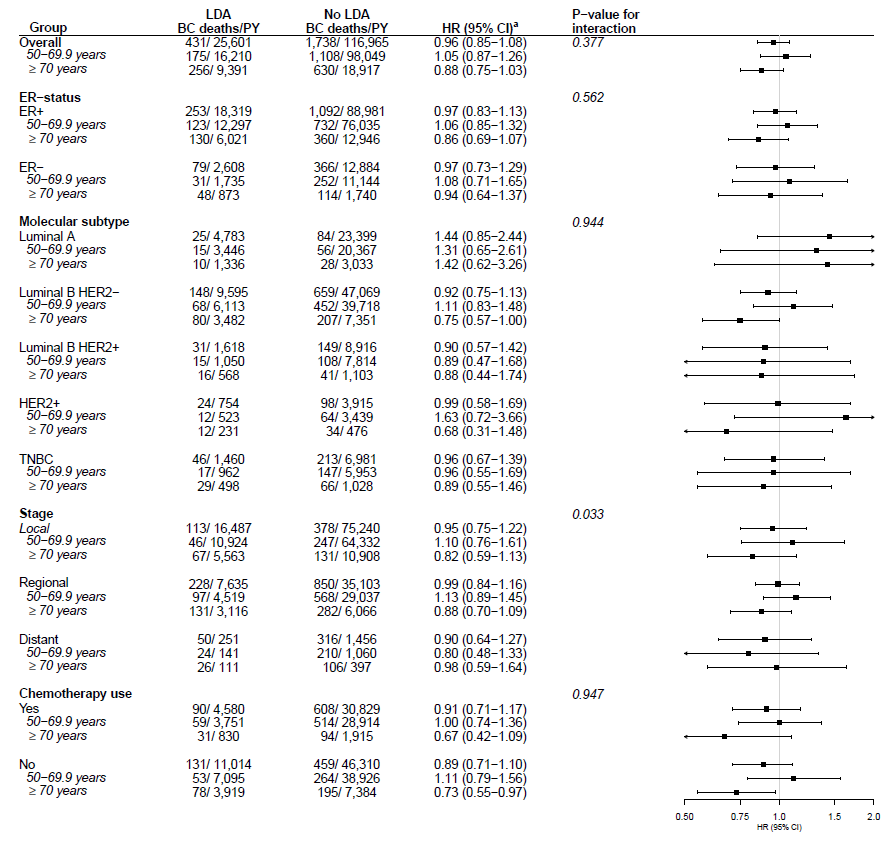


**Supplementary Figure 3** Association between post-diagnostic use (dispensation of ≥270 defined daily doses after diagnosis) of low-dose aspirin, compared to no use, and breast cancer-specific survival, Norway 2004–2017, by age, molecular subtype, stage, and use of chemotherapy

*Abbreviation:* Low-dose aspirin (LDA), breast cancer (BC), person-years (PY), hazard ratio (HR), confidence interval (CI), oestrogen receptor (ER), human epidermal growth factor receptor 2 (HER2), triple negative breast cancer (TNBC)

^a^ Adjusted for age at diagnosis, education, marital status, number of children, country of origin, stage, molecular subtype, histology, and post-diagnostic use of concomitant medications (statins, metformin, non-aspirin antiplatelets, non-metformin antidiabetics, non-steroidal anti-inflammatory drugs, beta-blockers, angiotensin converting enzyme inhibitors, angiotensin receptor blockers, calcium channel blockers, and diuretics). The stratified estimates are adjusted for all variables except for the specific stratification variable.

**
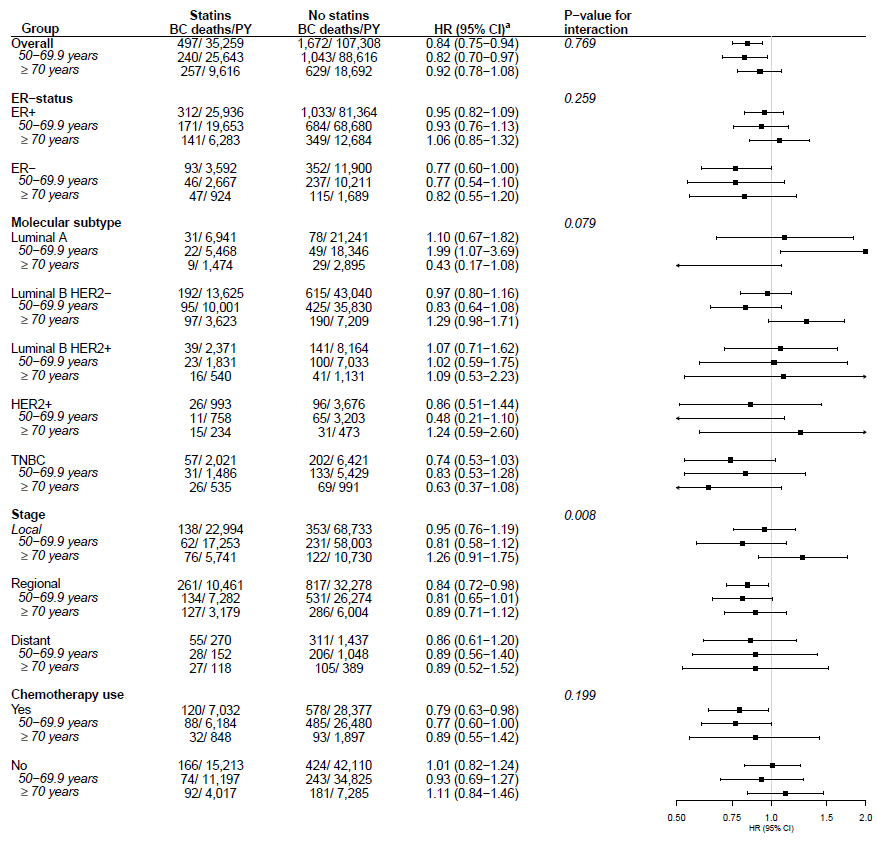
**

**Supplementary Figure 4** Association between post-diagnostic use (dispensation of ≥270 defined daily doses after diagnosis) of statins, compared to no use, and breast cancer-specific survival, Norway 2004–2017, by age, molecular subtype, stage, and use of chemotherapy

*Abbreviation:* Breast cancer (BC), person-years (PY), hazard ratio (HR), confidence interval (CI), oestrogen receptor (ER), human epidermal growth factor receptor 2 (HER2), triple negative breast cancer (TNBC)

^a^ Adjusted for age at diagnosis, education, marital status, number of children, country of origin, stage, molecular subtype, histology, and post-diagnostic use of concomitant medications (low-dose aspirin, non-aspirin antiplatelets, metformin, non-metformin antidiabetics, non-steroidal anti-inflammatory drugs, beta-blockers, angiotensin converting enzyme inhibitors, angiotensin receptor blockers, calcium channel blockers, and diuretics). The stratified estimates are adjusted for all variables except for the specific stratification variable.

**
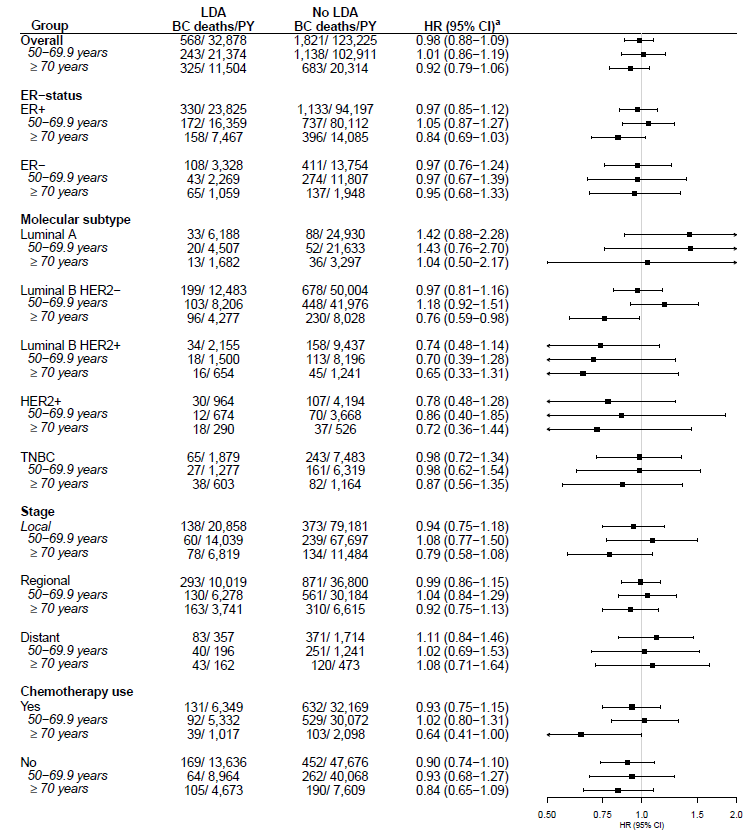
**

**Supplementary Figure 5** Association between post-diagnostic use (dispensation of ≥100 defined daily doses after diagnosis) of low-dose aspirin, compared to no use, and breast cancer-specific survival, Norway 2004–2018, by age, molecular subtype, stage, and use of chemotherapy

*Abbreviation:* Low-dose aspirin (LDA), breast cancer (BC), person-years (PY), hazard ratio (HR), confidence interval (CI), oestrogen receptor (ER), human epidermal growth factor receptor 2 (HER2), triple negative breast cancer (TNBC)

^a^ Adjusted for age at diagnosis, education, marital status, number of children, country of origin, stage, molecular subtype, histology, and post-diagnostic use of concomitant medications (statins, metformin, non-aspirin antiplatelets, non-metformin antidiabetics, non-steroidal anti-inflammatory drugs, beta-blockers, angiotensin converting enzyme inhibitors, angiotensin receptor blockers, calcium channel blockers, and diuretics). The stratified estimates are adjusted for all variables except for the specific stratification variable.


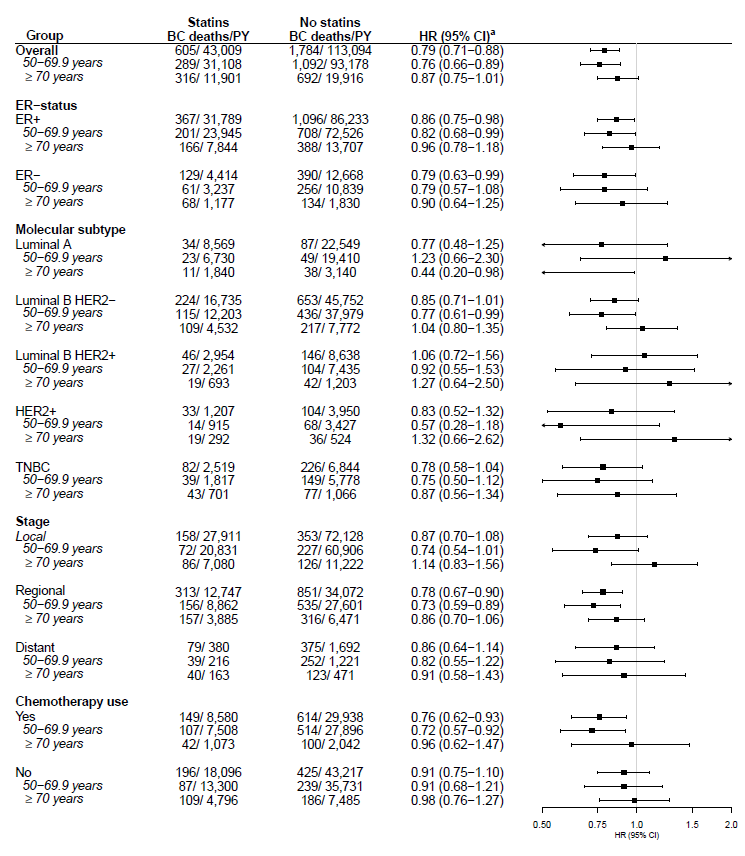


**Supplementary Figure 6** Association between post-diagnostic use (dispensation of ≥100 defined daily doses after diagnosis) of statins, compared to no use, and breast cancer-specific survival, Norway 2004–2018, by age, molecular subtype, stage, and use of chemotherapy

*Abbreviation:* Breast cancer (BC), person-years (PY), hazard ratio (HR), confidence interval (CI), oestrogen receptor (ER), human epidermal growth factor receptor 2 (HER2), triple negative breast cancer (TNBC)

^a^ Adjusted for age at diagnosis, education, marital status, number of children, country of origin, stage, molecular subtype, histology, and post-diagnostic use of concomitant medications (low-dose aspirin, non-aspirin antiplatelets, metformin, non-metformin antidiabetics, non-steroidal anti-inflammatory drugs, beta-blockers, angiotensin converting enzyme inhibitors, angiotensin receptor blockers, calcium channel blockers, and diuretics). The stratified estimates are adjusted for all variables except for the specific stratification variable.


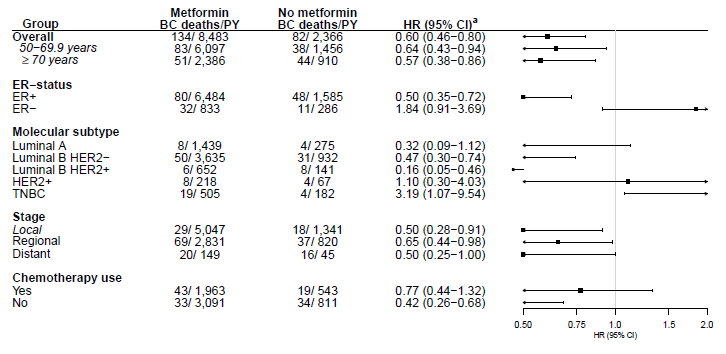


**Supplementary Figure 7** Association between post-diagnostic use (dispensation of ≥100 defined daily doses after diagnosis) of metformin, compared to use of non-metformin antidiabetics, and breast cancer-specific survival, Norway 2004–2018, by age, molecular subtype, stage, and use of chemotherapy

*Abbreviation:* Breast cancer (BC), person-years (PY), hazard ratio (HR), confidence interval (CI), oestrogen receptor (ER), human epidermal growth factor receptor 2 (HER2), triple negative breast cancer (TNBC)

^a^ Adjusted for age at diagnosis, education, marital status, number of children, country of origin, stage, molecular subtype, histology, and post-diagnostic use of concomitant medications (low-dose aspirin, non-aspirin antiplatelets, statins, non-steroidal anti-inflammatory drugs, beta-blockers, angiotensin converting enzyme inhibitors, angiotensin receptor blockers, calcium channel blockers, and diuretics). The stratified estimates are adjusted for all variables except for the specific stratification variable.

**
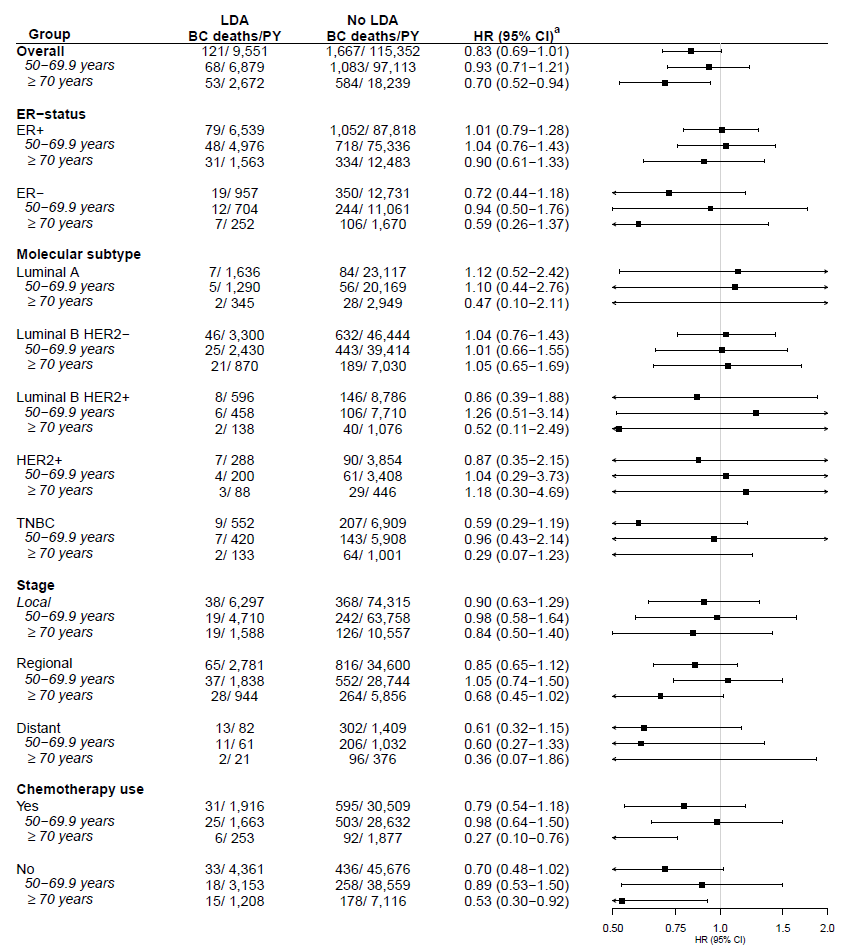
**

**Supplementary Figure 8** Association between post-diagnostic use (dispensation of ≥270 defined daily doses after diagnosis) of low-dose aspirin, compared to no use, and breast cancer-specific survival, Norway 2004–2017, by age, molecular subtype, stage, and use of chemotherapy. Including incident users only

*Abbreviation:* Low-dose aspirin (LDA), breast cancer (BC), person-years (PY), hazard ratio (HR), confidence interval (CI), oestrogen receptor (ER), human epidermal growth factor receptor 2 (HER2), triple negative breast cancer (TNBC)

^a^ Adjusted for age at diagnosis, education, marital status, number of children, country of origin, stage, molecular subtype, histology, and post-diagnostic use of concomitant medications (statins, metformin, non-aspirin antiplatelets, non-metformin antidiabetics, non-steroidal anti-inflammatory drugs, beta-blockers, angiotensin converting enzyme inhibitors, angiotensin receptor blockers, calcium channel blockers, and diuretics). The stratified estimates are adjusted for all variables except for the specific stratification variable.


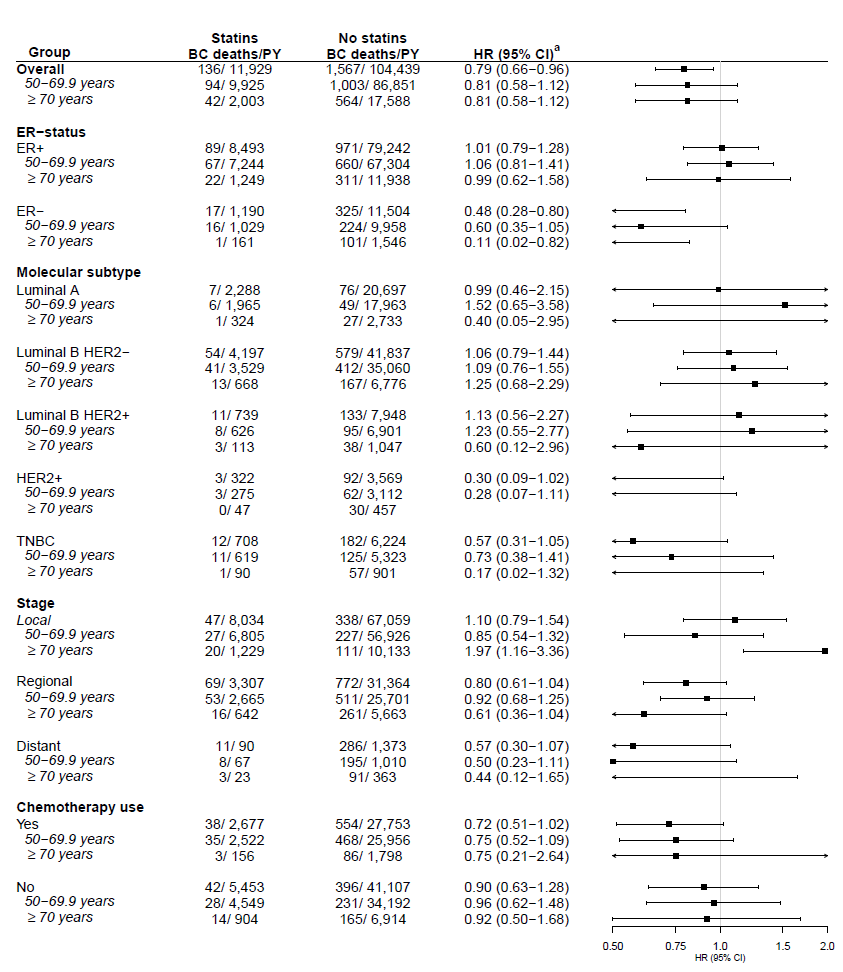


**Supplementary Figure 9** Association between post-diagnostic use (dispensation of ≥270 defined daily doses after diagnosis) of statins, compared to no use, and breast cancer-specific survival, Norway 2004–2017, by age, molecular subtype, stage, and use of chemotherapy. Including incident users only

*Abbreviation:* Breast cancer (BC), person-years (PY), hazard ratio (HR), confidence interval (CI), oestrogen receptor (ER), human epidermal growth factor receptor 2 (HER2), triple negative breast cancer (TNBC)

^a^ Adjusted for age at diagnosis, education, marital status, number of children, country of origin, stage, molecular subtype, histology, and post-diagnostic use of concomitant medications (low-dose aspirin, non-aspirin antiplatelets, metformin, non-metformin antidiabetics, non-steroidal anti-inflammatory drugs, beta-blockers, angiotensin converting enzyme inhibitors, angiotensin receptor blockers, calcium channel blockers, and diuretics). The stratified estimates are adjusted for all variables except for the specific stratification variable.


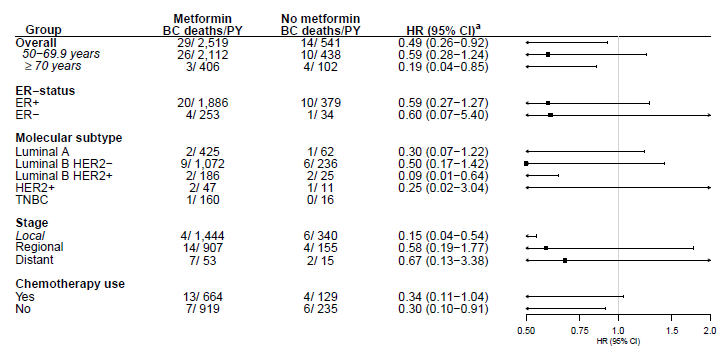


**Supplementary Figure 10** Association between post-diagnostic use (dispensation of ≥270 defined daily doses after diagnosis) of metformin, compared to use of non-metformin antidiabetics, and breast cancer-specific survival, Norway 2004–2017, by age, molecular subtype, stage, and use of chemotherapy. Including incident users only

*Abbreviation:* Breast cancer (BC), person-years (PY), hazard ratio (HR), confidence interval (CI), oestrogen receptor (ER), human epidermal growth factor receptor 2 (HER2), triple negative breast cancer (TNBC)

^a^ Adjusted for age at diagnosis, education, marital status, number of children, country of origin, stage, molecular subtype, histology, and post-diagnostic use of concomitant medications (low-dose aspirin, non-aspirin antiplatelets, statins, non-steroidal anti-inflammatory drugs, beta-blockers, angiotensin converting enzyme inhibitors, angiotensin receptor blockers, calcium channel blockers, and diuretics). The stratified estimates are adjusted for all variables except for the specific stratification variable.

**
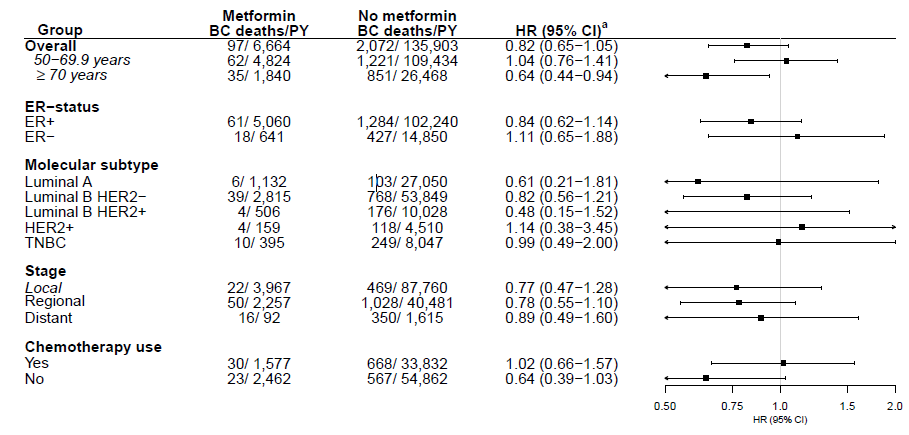
**

**Supplementary Figure 11** Association between post-diagnostic use (dispensation of ≥270 defined daily doses after diagnosis) of metformin, compared to no use, and breast cancer-specific survival, Norway 2004–2017, by age, molecular subtype, stage, and use of chemotherapy.

*Abbreviation:* Breast cancer (BC), person-years (PY), hazard ratio (HR), confidence interval (CI), oestrogen receptor (ER), human epidermal growth factor receptor 2 (HER2), triple negative breast cancer (TNBC)

^a^ Adjusted for age at diagnosis, education, marital status, number of children, country of origin, stage, molecular subtype, histology, and post-diagnostic use of concomitant medications (low-dose aspirin, non-aspirin antiplatelets, statins, non-steroidal anti-inflammatory drugs, beta-blockers, angiotensin converting enzyme inhibitors, angiotensin receptor blockers, calcium channel blockers, and diuretics). The stratified estimates are adjusted for all variables except for the specific stratification variable.

**
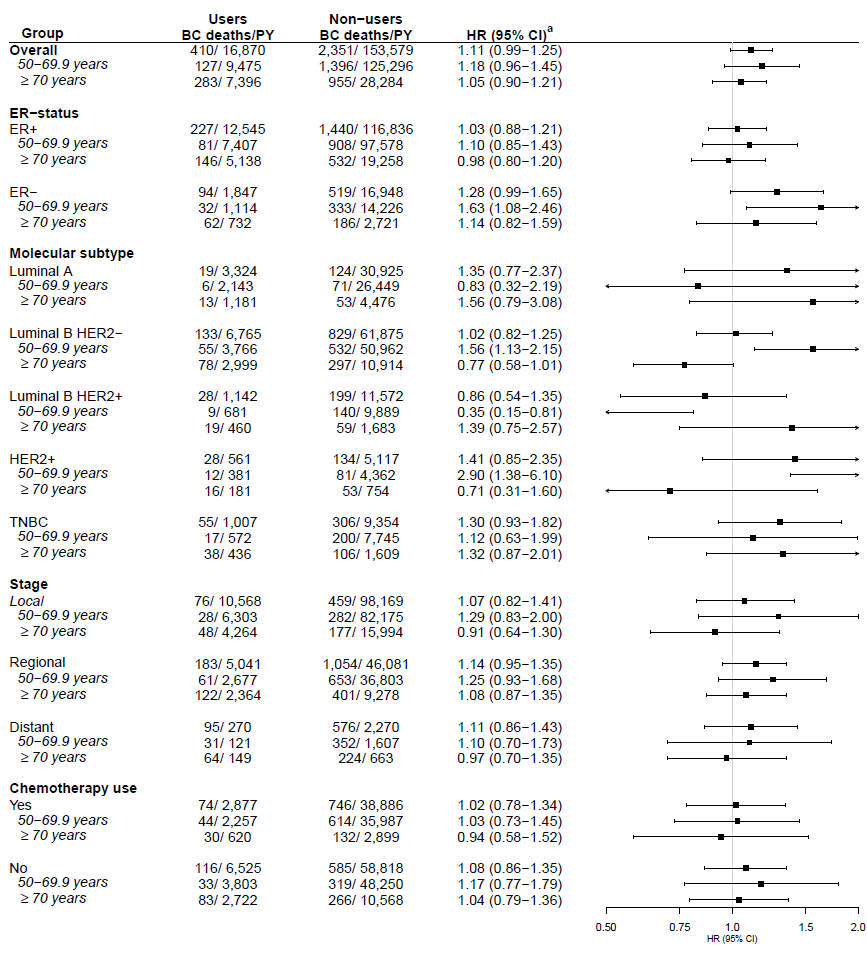
**

**Supplementary Figure 12**Association between peri-diagnostic use (at least one dispensation within three months prior to the diagnosis) of low-dose aspirin, compared to no use, and breast cancer-specific survival, Norway 2004–2018, by age, molecular subtype, stage, and use of chemotherapy.

*Abbreviation:* Breast cancer (BC), person-years (PY), hazard ratio (HR), confidence interval (CI), oestrogen receptor (ER), human epidermal growth factor receptor 2 (HER2), triple negative breast cancer (TNBC)

^a^ Adjusted for age at diagnosis, education, marital status, number of children, country of origin, stage, molecular subtype, histology, and peri-diagnostic use of concomitant medications (statins, metformin, non-aspirin antiplatelets, non-metformin antidiabetics, non-steroidal anti-inflammatory drugs, beta-blockers, angiotensin converting enzyme inhibitors, angiotensin receptor blockers, calcium channel blockers, and diuretics). The stratified estimates are adjusted for all variables except for the specific stratification variable.


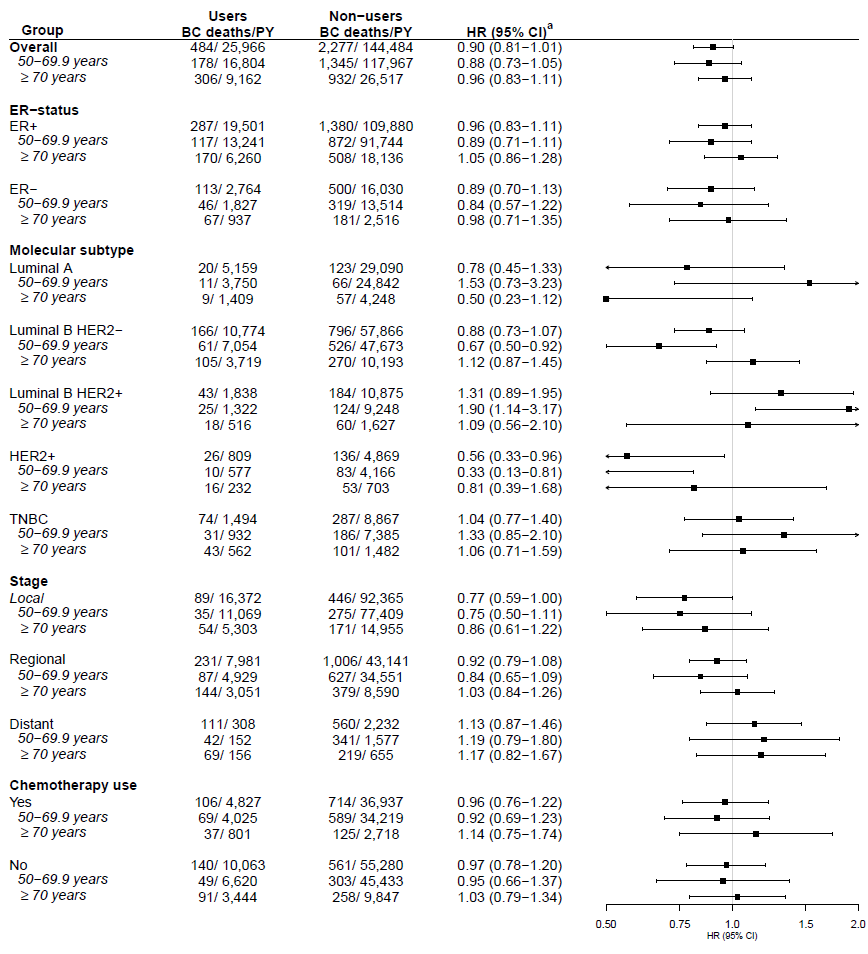


**Supplementary Figure 13** Ass1ciation between peri-diagnostic use (at least one dispensation within three months prior to the diagnosis) of statins, compared to no use, and breast cancer-specific survival, Norway 2004–2018, by age, molecular subtype, stage, and use of chemotherapy.

*Abbreviation:* Breast cancer (BC), person-years (PY), hazard ratio (HR), confidence interval (CI), oestrogen receptor (ER), human epidermal growth factor receptor 2 (HER2), triple negative breast cancer (TNBC)

^a^ Adjusted for age at diagnosis, education, marital status, number of children, country of origin, stage, molecular subtype, histology, and peri-diagnostic use of concomitant medications (low-dose aspirin, non-aspirin antiplatelets, metformin, non-metformin antidiabetics, non-steroidal anti-inflammatory drugs, beta-blockers, angiotensin converting enzyme inhibitors, angiotensin receptor blockers, calcium channel blockers, and diuretics). The stratified estimates are adjusted for all variables except for the specific stratification variable.


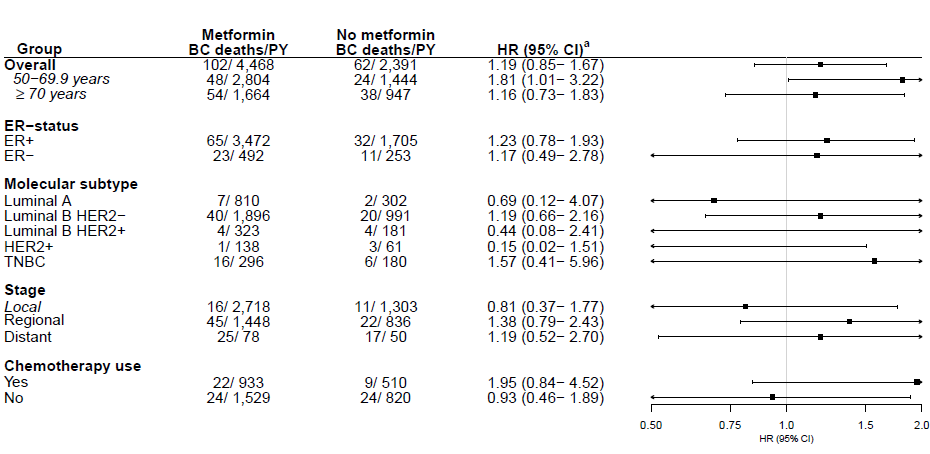


**Supplementary Figure 14** Association between peri-diagnostic use (at least one dispensation within three months prior to the diagnosis) of metformin, compared to use of non-metformin antidiabetics, and breast cancer-specific survival, Norway 2004–2018, by age, molecular subtype, stage, and use of chemotherapy.

*Abbreviation:* Breast cancer (BC), person-years (PY), hazard ratio (HR), confidence interval (CI), oestrogen receptor (ER), human epidermal growth factor receptor 2 (HER2), triple negative breast cancer (TNBC)

^a^ Adjusted for age at diagnosis, education, marital status, number of children, country of origin, stage, molecular subtype, histology, and peri-diagnostic use of concomitant medications (low-dose aspirin, non-aspirin antiplatelets, statins, non-steroidal anti-inflammatory drugs, beta-blockers, angiotensin converting enzyme inhibitors, angiotensin receptor blockers, calcium channel blockers, and diuretics). The stratified estimates are adjusted for all variables except for the specific stratification variable.
